# Adipokine RBP4 drives ovarian cancer cell migration

**DOI:** 10.1186/s13048-018-0397-9

**Published:** 2018-04-11

**Authors:** Yanyan Wang, Yilin Wang, Zhenyu Zhang

**Affiliations:** 1 Department of Gynaecology and Obstetrics, Beijing Chaoyang Hospital, Capital Medical University, No. 8 South Road, workers’ Stadium, Chaoyang District, Beijing, 100020 China; 2 The First Affiliated Hospital of Jinzhou Medical University, No.2, people’s street, Jinzhou, 121001 China

**Keywords:** Ovarian cancer, Obesity, RBP4, Retinol acid, Migration

## Abstract

**Background:**

Obesity has been linked to several types of cancers including ovarian cancer. Retinol binding protein 4 (RBP4) is an adipokine that drives the development of hyperinsulinemia and type II diabetes in obesity patients and animals. Previously, we have identified RBP4 as a serum marker for ovarian cancer. Here we further explored the consequence of RBP4 upregulation in ovarian cancer cells and its molecular mechanism.

**Results:**

Our results show that RBP4 is overexpressed in ovarian cancer cells to the same extent as in adipose tissues. The overexpression of RBP4 in ovarian cancer cells promotes cancer cell migration and proliferation. At molecular level, cancer progression factors MMP2 and MMP9 are induced in response to RBP4 overexpression. We further investigated which signaling pathways are utilized by RBP4 to activate ovarian cancer cell migration. We found RhoA/Rock1 pathway is turned on and CyclinD1 is upregulated in RBP4 overexpressed cells. Inhibition of RhoA/Rock1 pathway reduces the RBP4-induced MMP2 and MMP9 expression. The RBP4 action is depend on its associated ligand vitamin A/retinol acid (RA) and possibly involves similar pathways as for conferring insulin resistance. Moreover, we show that knockdown of RBP4 significantly reduce cancer cell migration and proliferation as well as expressions of oncogenic factors.

**Conclusions:**

Our results indicated that RBP4 can drive ovarian cancer cell migration and proliferation via RhoA/Rock1 and ERK pathway. It suggests that RBP4 act as a oncogene in ovarian cancer cells. Thus, RBP4 could be a molecular bridge between obesity and cancers and a potential target for treating obese cancer patients.

## Background

Obesity is a well-established cancer risk factor and its occurrence is strongly associated with several types of cancers, including breast, colon, endometrial, ovarian, gastric, pancreatic and liver cancers [1, 2]. However, the molecular mechanisms that link obesity and cancers remain largely elusive. Identifying metabolites and secreted factors that connect increased fat mass to tumorigenesis is one of the central questions.

Retinol binding protein 4 (RBP4) is secreted by liver and adipose tissues [3, 4]. RBP4 acts as the major transporter for vitamin A/retinol acid (RA) in serum [3]. Under normal physical conditions, RA bound RBP4 circulates together with transthyretin (TTR) as a holo-RBP-TTR complex [5]. Upon arrival, RA can either enter the targeted cell by passive diffusion or active transportation by Stimulated by RA 6 (STRA6) [6–10].

Besides its transportation function, RBP4 has recently been recognized as an adipokine [4]. Cumulative evidences showed that overexpression of RBP4 from adipose tissues promote hyperinsulinemia and type II diabetes [11–15]. Several pathways have been identified mediate RBP4 signaling [16]. RA and its oxidative products can activate retinoic acid receptors and retinoid X receptors and promote glucose production in liver [17]. RBP4 with RA can activate STRA6, which will then recruit and activate Janus kinase and the transcription factors STAT3 or STAT5 [18]. RBP4, independent of RA and STRA6, could promote pro-inflammatory responses possibly through pathways involving c-Jun N-terminal protein kinase (JNK)1, JNK2, or Toll-like receptor [19].

Ovarian cancer is the most lethal type of gynecological cancer in the world [20]. Ovarian cancers have a high occurrence rate in obesity peoples and obesity has been shown to promote ovarian cancer metastatic [21]. Recently, we found that RBP4 level is highly upregulated in ovarian cancer serum samples [22]. Overexpression of RBP4 had also been reported in liver, bone, and colon cancer cells [23–27]. However, the consequence of RBP4 overexpression on cancers and the mechanism of action of RBP4 in cancers are not clear.

We here investigated whether RBP4 is a tumorgenic factor that connects obesity and ovarian cancer. Our data showed that RBP4 was up-regulated in ovarian cancer cells and overexpression of RBP4 promoted cancer cell migration. The MMP-2 and MMP-9, key factors in cancer metastasis, were induced by RBP4 overexpression. We further identified RhoA/Rock1 pathway as mediators for RBP4 action. RhoA and Rock1 were overexpressed in response to RBP4 and inhibition of RhoA/Rock1 reduced MMP-2 and MMP-9 expression. The RBP4 action was dependent on its associated ligand RA. Moreover, knockdown of RBP4 greatly reduced cancer migration. Our data not only established RBP4 as a direct linkage between obesity and ovarian cancer, but also suggested RBP4 was a possible target for cancer treatment, especially in those associated with obesities.

## Methods

### Study samples

This study was approved by the Medical Ethical Committee of Beijing Chaoyang Hospital (Beijing, China). The written informed consents were obtained from all the participants enrolled in the study. Specimens were sampled from patients undergoing surgery for ovarian carcinoma or benign ovarian tissues at the Beijing Chaoyang Hospital.

All procedures were approved by the Animal Care and Use Committee of Beijing Chaoyang Hospital (Beijing, China). All experiment methods were performed in accordance with the relevant guidelines and regulations. In brief, healthy specific-pathogen-free (SPF) male SD rats were purchased from the Vital River. All rats were preserved under standard housing laboratory conditions. After one week of adaptation to the diet and the new environment, female SD rats were divided into two diet groups: the normal control (NC) group fed ad libitum a standard rodent chow, the high-fat (HF) group fed ad libitum a high-fat chow. After six weeks to induce obesity, the ovarian tissues were obtained after euthanasia.

### Reagents

Antibodies against RBP4 (#ab109193), actin (#ab8226), RhoA (#ab187027), p-RhoA (#ab41435), ROCK1 (#ab45171), Erk (#ab54230), p-Erk (#ab51100), Cyclin D1 (#ab134175), MMP2 (#ab37150), MMP9 (#ab38898) were obtained from Abcam, USA. ROCK1 inhibitor Y27632 was purchased from Sigma, USA. All primers for qPCR were ordered from Invitrogen (Shanghai, China).

Human ovarian cancer cell line A2780 was obtained from Prof. Haiteng Deng laboratory [28, 29]. SKOV3 was preserved in our lab [30]. Cells were maintained in DMEM medium in incubator with 5% CO_2_ at 37 °C.

### Cell transfection

To upregulate the expression of RBP4, the human RBP4 full length cDNA was amplified and inserted into the pCMV-Flag vector, and a scramble sequence was inserted into the pCMV-flag vector as the control vector. To knock down the expression of RBP4, a RBP4 siRNA was designed and obtained from Jima Inc. (Shanghai, China). For transfection, the cells were seeded into 6-well plates. When cell confluency reached 50%, RBP4 siRNA or RBP4-pCMV-Flag was transfected into the cells using lipo2000 according to the manufacturer’s instructions.

### Western blotting

Cells were lysed in RIPA buffer with protease and phosphatase inhibitor cocktail. Equal amount of protein samples was loaded onto 12% SDS-PAGE and was then transferred to PVDF membranes. After blocking with 5% BSA for 1 h at room temperature, the membranes were incubated with primary antibodies at 4 °C overnight. Then, the membranes were incubated with horseradish peroxidase-conjugated secondary antibody (from Zhongshanjinqiao, China) for 1 h at room temperature. The protein bands were visualized by ChemiDoc XRS+ (BioRad, USA). Data analysis was done using Quantity one.

### Quantitative reverse transcription- polymerase chain reaction (qRT-PCR)

Total RNA was isolated from the cells using trizol method. RNA was reverse transcribed using the PrimeScript RT Master Mix (B-Belife, China) according to the manufacturer’s instructions. The PCR amplifications were performed using SYBR Premix Ex Taq II (B-Belife, China). The expression level of each sample was internally normalized against that of the glyceraldehyde 3-phosphate dehydrogenase (GAPDH). The relative quantitative value was calculated using 2^−ΔΔCt^ method. Each experiment was performed in triplicate. The primers used in real-time PCR were as follow: RBP4 F: AGGAGAACTTCGACAAGGCTC; RBP4 R: GAGAACTCCGCGACGATGTT; GAPDH F: GGAGCGAGATCCCTCCAAAAT; GAPDH R: GGCTGTTGTCATACTTCTCATGG; RHOA F: AGCCTGTGGAAAGACATGCTT; RHOA R: TCAAACACTGTGGGCACATAC; Rock1 F: AACATGCTGCTGGATAAATCTGG; Rock1 R: TGTATCACATCGTACCATGCCT; cyclinD1(CCND1) F: GCTGCGAAGTGGAAACCATC; cyclinD1(CCND1) R: CCTCCTTCTGCACACATTTGAA; ERK1 F: CTACACGCAGTTGCAGTACAT; ERK1 R: CAGCAGGATCTGGATCTCCC; MMP2 F: TACAGGATCATTGGCTACACACC; MMP2 R: GGTCACATCGCTCCAGACT; MMP9 F: TGTACCGCTATGGTTACACTCG; MMP9 R: GGCAGGGACAGTTGCTTCT.

### Immunohistochemistry

The expression of RBP4 was assessed using immunohistological staining as described previously [20]. Briefly, tissue samples were fixed and cut to 5 *μ*m thick. RBP4 antibody was applied on the sections for 30 min and incubated overnight at 4 °C then shaking at room temperature for 30 min. Antibody binding was amplified using biotin and streptavidin HRP for 10 min each and the complex was visualized using DAB. ALL sections were assessed microscopically for positive DAB staining. The immunostained sections were examined under microscopy and the expression level of RBP4 was scored on the basis of the intensity of staining.

### In vitro migration assay

A 24-well Transwell chamber (Corning, #3422, USA) was used to examine the invasive ability of the ovarian cancer cells. Cells were suspended in DMEM medium and were added into the upper Transwell chamber. The lower Transwell chamber was filled with DMEM medium supplemented with 10% FBS. After incubation of 16 h at 37 °C, the non-migrated cells were removed with a sterile cotton swab, and the migrated cells were stained with 0.1% crystal violet for 20 min at room temperature. The numbers of cells were calculated under a light microscope in five random fields.

### Proliferation assay

Cell proliferation was determined by MTT assay. Cells were seeded at 1000 cells/well in a 96-well plate. After incubation for indicated time, MTT was added into the plate incubated for 4 h. The optical density (OD) was measured 490 nm at designated time.

### Cell cycle analysis

Cell cycle distribution was analyzed by PI staining and flow cytometry. The 1 × 10^5^ cells / well were seeded in 6-well plates. The cells were then harvested, fixed with 70% ice cold ethanol, and stored at 4 °C until analysis. After fixation, the cells were washed twice with cold phosphate-buffered saline (PBS) and centrifuged, following which the supernatants were removed. The pellet was resuspended and stained with PBS containing 50 μg/ml PI and 100 μg/ml RNaseA for 20 min in the dark. The cell cycle data were analyzed using Modifit software.

### Statistical analysis

All the continuous variables were expressed as average ± standard deviation (SD). Student’s t-test was used for the difference analysis. A *P* value of more than 0.01 was considered as statistical significance. Graphpad 5.0 software was used for all the statistical analyses.

## Results

### Expression of RBP4 in ovarian cancer tissues and obesity tissues

**Fig. 1:**
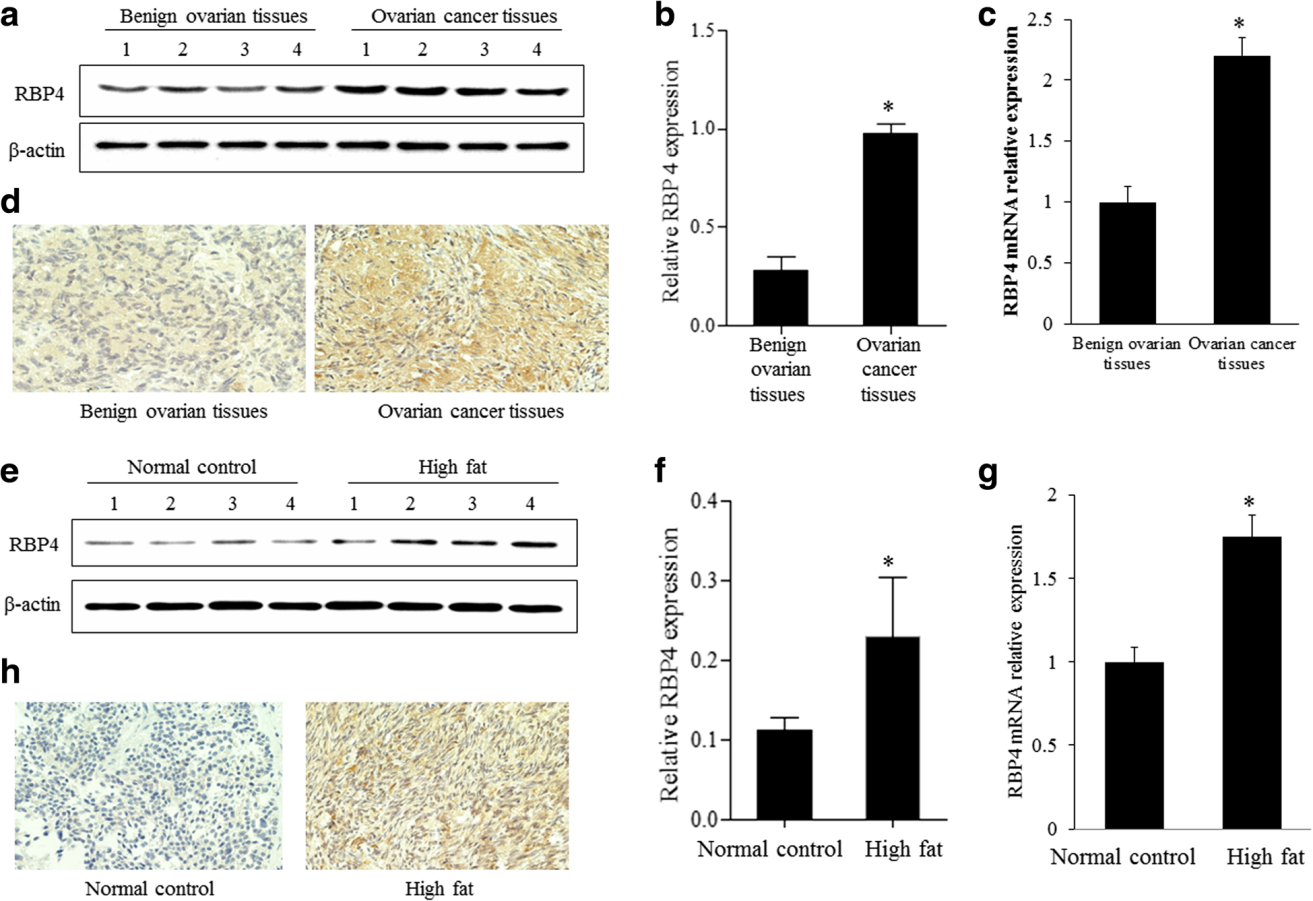
Expression of RBP4 in ovarian cancers and high fat group. **a**, **b**, **e** and **f**. Western blotting analysis of RBP4 in control, ovarian cancer group and high fat group. **c** and **g**. qPCR analysis of RBP4 expression in control, ovarian cancer group and high fat group. **d** and **h**. Immunostaining of RBP4 in control, ovarian cancer group and high fat group. *, *p* < 0.01

We first detected the RBP4 expression levels in ovarian cancer tissues. Western blot results showed that the RBP4 protein was upregulated by nearly 4-fold in ovarian cancer tissues comparing to the benign ovarian tissues (Fig. 1a-b). The higher expression of RBP4 was further verified by qRT-PCR experiment (Fig. 1c) and immunostaining (Fig. 1d). The mRNA level of RBP4, as revealed by qRT-PCR, was twofold higher in ovarian cancer tissues comparing to the benign ovarian tissues (Fig. 1c). The RBP4 level in cancer tissues, shown in brown, was significantly increased comparing to the benign ovarian tissues, which only exhibited weak staining (Fig. 1d). As a control, we created obese rat model by fed with a high-fat group rats and measured the expression level of RBP4 in ovarian tissues. Similarly as in previous report [4], the RBP4 level was elevated in ovarian tissues from the high fat (HF) group compared to the normal control (NC) group (Fig. 1e-h). The extent of RBP4 overexpression was comparable in ovarian cancer cells and in adipose tissues.

### RBP4 promotes migration and proliferation of ovarian cancer cells

**Fig. 2:**
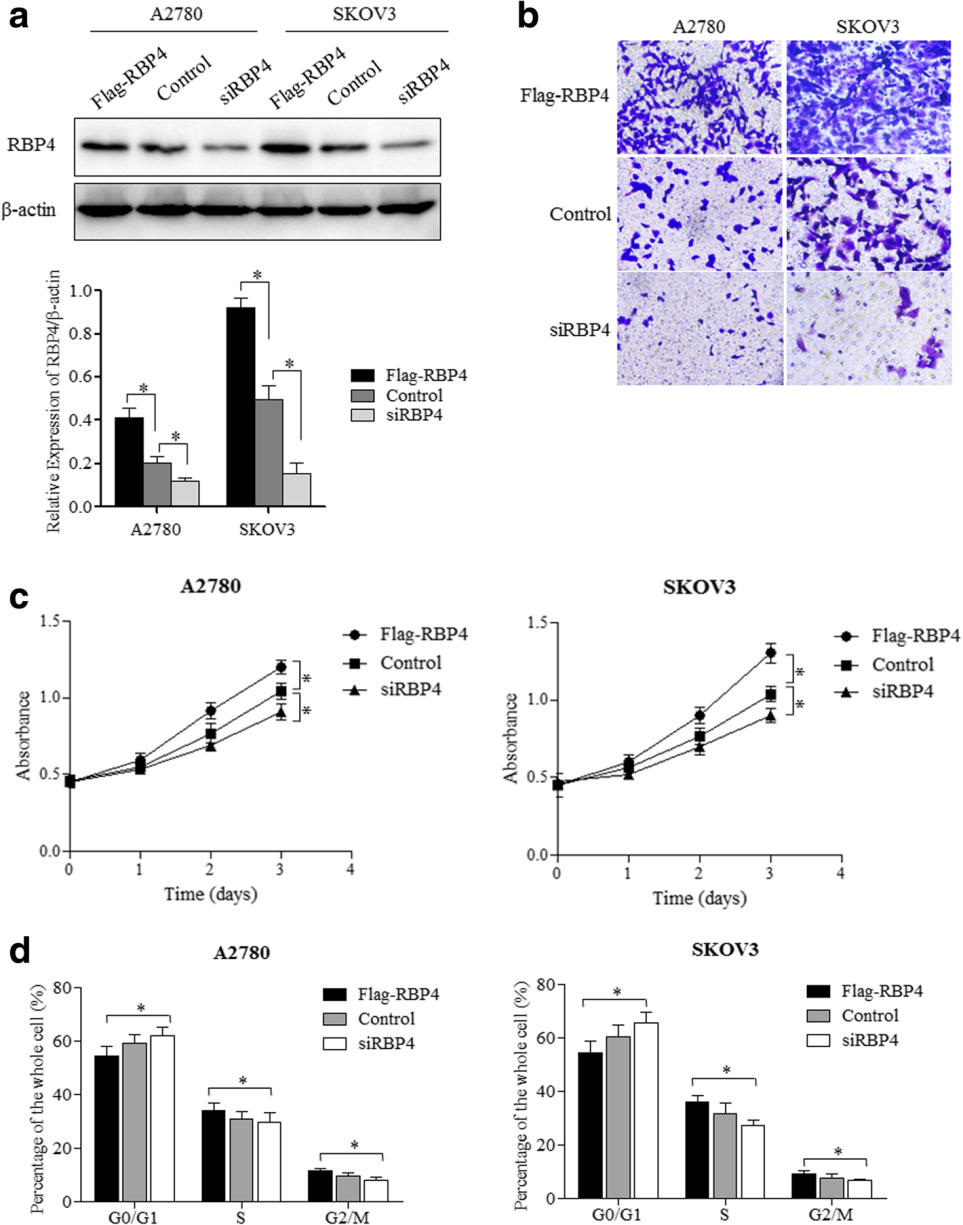
RBP4 promotes ovarian cancer cell migration and proliferation. **a**. Western blot analysis of RBP4 levels in cells with Flag-RBP4 overexpression, RBP4 knockdown and control cells. **b**. Cell migration assays of RBP4 overexpression, control and RBP4 knockdown cells. **c**. Cell proliferation profile of cells with RBP4 overexpression, control and RBP4 knockdown cells. **d**. Cell cycle distribution of cells with Flag-RBP4 overexpression, control and RBP4 knockdown cells. *, *p* < 0.01

We used ovarian cancer cell line A2780 and SKOV3 to test the effects of RBP4 expression on ovarian cancer. Firstly, we confirmed the effect of RBP4 overexpression and knocked down in A2780 and SKOV3 cells (Fig. 2a). Then the transwell migration assays showed that RBP4 overexpression can greatly enhance cancer cell migration in both cell lines (Fig. 2b). In contrast, cancer cells were less mobile when RBP4 was knocked down with siRNA (Fig. 2b). We then carried out proliferation assay to explore the effect of RBP4 expression on cell proliferation in A2780 and SKOV3 cells. The results showed that ovarian cancer cells with RBP4 overexpression grows faster than control cells, while the RBP4 knockdown inhibited cell proliferation (Fig. 2c). Finally, we analyzed the cell cycle distribution with respect to RBP4 expression. More cells were in S and G2/M phase when RBP4 overexpressed (Fig. 2d). Collectively, these results indicated that RBP4 promotes migration and proliferation of ovarian cancer cells.

### RBP4 induces migration-related genes expression in ovarian cancer cells

**Fig. 3:**
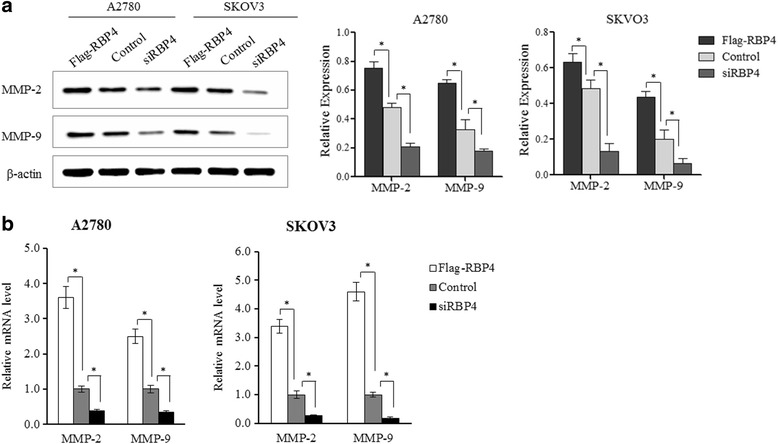
MMP2 and MMP9 expression is elevated by RBP4. **a**. Western blot analysis of MMP2 and MMP9 levels in cells with Flag-RBP4 overexpression, RBP4 knockdown and control cells. **b**. qPCR analysis of MMP2 and MMP9 levels in cells with Flag-RBP4 overexpression, RBP4 knockdown and control cells. *, *p* < 0.01

We have shown that RBP4 overexpression can greatly stimulate ovarian cancer cell migration. Then, we tested the expression level of MMP2 and MMP9, which are essential for cancer metastasis [31]. Both protein and mRNA levels of MMP2 and MMP9 were elevated when RBP4 overexpressed in SKOV3 and A2780 cells (Fig. 3). The observation further confirmed the effect of RBP4 expression on cancer cell migration.

### RBP4 activates RhoA/Rock1 pathways and cyclin D1

**Fig. 4:**
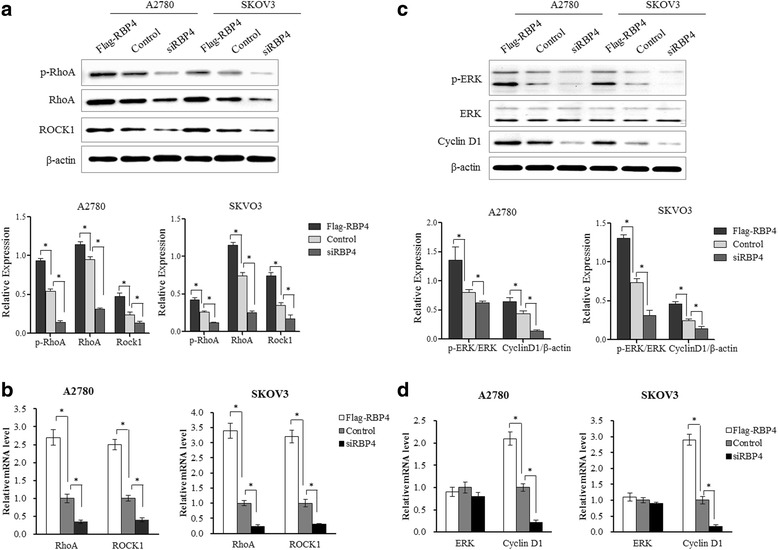
Expression of major tumorigenic signaling factors in response to RBP4 expression. **a**. Western blotting analysis of RhoA, p-RhoA and ROCK1 levels in cells with Flag-RBP4 overexpression, RBP4 knockdown and control cells. **b**. qPCR analysis of RhoA and ROCK1 levels in cells with Flag-RBP4 overexpression, RBP4 knockdown and control cells. **c**. Western blotting analysis of ERK, p-ERK and CyclinD1 levels in cells with Flag-RBP4 overexpression, RBP4 knockdown and control cells. **d**. qPCR analysis of ERK and CyclinD1 levels in cells with Flag-RBP4 overexpression, RBP4 knockdown and control cells. *, *p* < 0.01

To explore how RBP4 exerts its effect on cancer cells, we tested the expression level of several key players in tumorigenesis related signaling. RBP4 overexpression stimulated the expression of both RhoA and Rock1. In RBP4 overexpression groups, the RhoA and Rock1 levels were elevated at both mRNA and protein level (Fig. 4a, b). Moreover, the p-RhoA level was also increased, revealed by western blotting. Similarly, the Cyclin D1 expression was promoted at both mRNA and protein level. Previously, overexpressed phospho-ERK triggerd ovarian cancer cell migration [32, 33]. Although the ERK expression level remained unchanged upon RBP4 overexpression, the phosphorylated ERK had been elevated by RBP4 overexpression (Fig. 4c, d). Interestingly, suppression of RBP4 expression significantly reduced the level of RhoA/Rock1 as well as Cyclin D1 comparing to control group (Fig. 4).

### RBP4 action is partially dependent on RhoA1/rock pathway

**Fig. 5:**
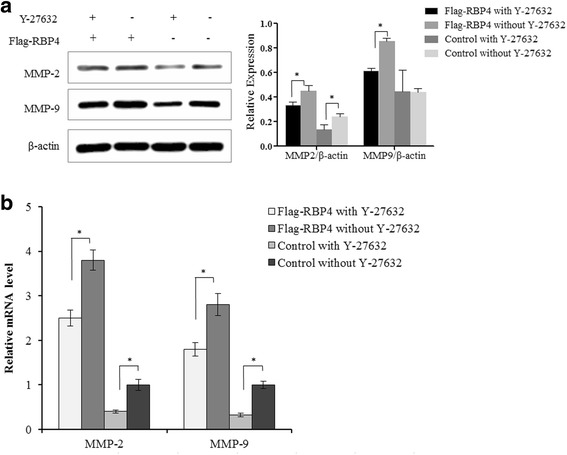
RBP4 induced MMP-2 and MMP-9 overexpression is partially dependent on RhoA/Rock1 pathway. **a**. Effect of ROCK1 inhibitor Y-27632 on MMP2 and MMP9 expression examined by western blotting. **b**. Effect of ROCK1 inhibitor Y-27632 on MMP2 and MMP9 expression examined by qPCR. The cells were treated with Y-27632 (10 μm) for 24 h before harvesting. *, p < 0.01

Rock1 inhibitor Y-27632 had been shown to effectively inhibit Rock1 and its associated pathways [34]. To test if RBP4 induced cancer cell migration is RhoA/Rock1 dependent, we added Y-27632 to our RBP4 overexpression cells. Y-27632 can effectively reduce the MMP-2/MMP9 expression level even when RBP4 overexpressed. However, the level of MMP2/MMP9 with Y-27632 was still higher than control group. The results indicated that the RBP4 action was partially depending on Rock1 pathway (Fig. 5a and b).

### RBP4 action is dependent on RA

**Fig. 6:**
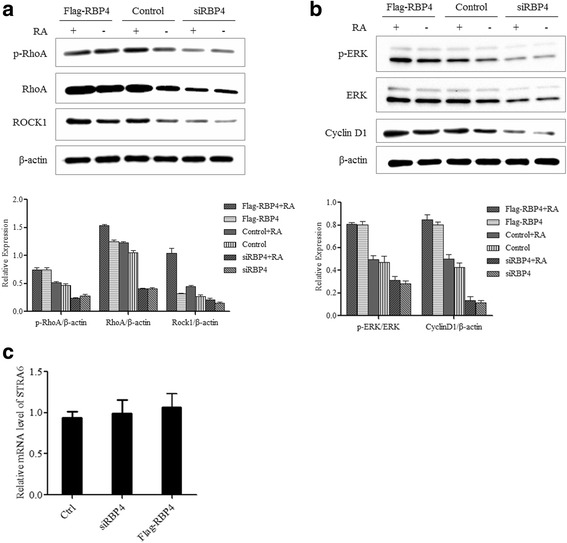
RBP4 action is dependent on RA. **a**. Western blot analysis of RhoA, p-RhoA and ROCK1 levels in cells with or without RA. **b**. Western blot analysis of ERK, p-ERK and CyclinD1 levels in cells with or without RA. **c**. qPCR analysis of STRA6 levels in cells with Flag-RBP4 overexpression, RBP4 knockdown and control cells. *, *p* < 0.01

RBP4 is a major RA transport protein [3]. We tested whether the RBP4 effect on ovarian cancer cells is RA dependent. In the absence of RA, the RBP4 overexpression had moderate stimulation on RhoA/Rock1 and Cyclin D1 expression (Fig. 6a, b). When RA was added, the RBP4 effect was stimulated. As a control, RA had little effect when RBP4 is suppressed. RA was partially transported through membrane transporter STRA6 and RBP4-RA complex had been shown to activate STRA6 and its associated signaling pathways [6–10]. We tested the expression level of STRA6 in ovarian cancer cells with qRT-PCR. The STRA6 mRNA level stayed the same in either RBP4 overexpression or knockdown cells (Fig. 6c).

## Discussion

Obese patients are associated with high cancer risk, poor prognosis and reduced response to anti-cancer therapies [1, 2]. Obesity is intrinsically linked with metabolic syndrome that can indirectly promote cancers as metabolic reprogramming is a hallmark of cancer. Especially for ovarian cancer, obesity has been shown to promote cancer metastasis [21]. Besides that obesity can indirectly affect cancer metastasis through increasing lipogenesis, enhancing vascularity, and decreasing infiltration [21]. Here, we established a direct molecular linkage between adipokine RBP4 and ovarian cancer. RBP4 is a well-established obesity factors that is overexpressed by adipose tissues [4]. We proved that high level of RBP4 can stimulate migration and proliferation of ovarian cancer cells. The overexpression of RBP4 stimulated the expression of matrix metalloproteinase MMP-2 and MMP-9, which degraded extracellular matrix and enabled cancer cells migration. Moreover, RBP4 highly expressed in ovarian cancer cells and high level of RBP4 had been documented in ovarian patient’s serum samples [22]. We thus proposed that high level of RBP4, either from adipose tissues or cancer tissues, can promote cancer metastasis and obesity signaling, vice versa. Although currently lack of clinical data, it would be interesting to survey if ovarian cancer patients have a high rate of obesity and insulin resistance.

RBP4 and its associated RA have been shown to trigger several downstream pathways to confer insulin resistance [16–19]. The pathways could also be shared in promoting cancer metastasis. The effect of RBP4 on tumor metastasis is RA dependent. RA has broad metabolic roles including stimulating lipogenesis [35], which has been shown to promote ovarian cancer metastasis [21]. It has been documented that RBP4 bound RA, but not apo RBP4 can induce signaling of STRA6 [18]. The STRA6 expression and its signaling has been proposed drive oncogenic transformation of cancer cells [34]. STRA6 expressed in the ovarian cancer cell lines, although its level was not affected by RBP4. STRA6 could mediate, at least part of the RBP4 effect. Circulating RBP4-RA was associated with their partner protein TTR [5], which inhibited the RBP4-RA triggered STRA6 signaling [7]. To our knowledge, there is no correlation of expression level of TTR with ovarian cancer. It is quite likely that when RBP4 overexpressed, the TTR was not enough to block STRA6 signaling even STRA6 level remained unchanged. On the other hand, RBP4 itself without RA can still promote metastasis, although to a lesser degree. It has been shown RBP4, independent of RA and STRA6, can induce pro-inflammation reaction [19].

Adding to the existing knowledge, we showed that RhoA/Rock1 pathway was turned on in response to RBP4 overexpression. RhoA/Rock1 pathway played pivot roles in cell morphogenesis, adhesion, and motility and was often activated in malignant cancers [36]. Previous reports had shown that inhibition of RhoA/Rock1 suppressed MMP-2 and MMP-9 action [37–39]. Consistently, we observed that inhibiting RhoA/Rock1 pathway with Rock1 inhibitor Y-27632 can reduce RBP4 induced MMP-2 and MMP-9 overexpression, indicating that the migration effect of RBP4 was mediated by RhoA/Rock1 pathway. However, how RBP4 activated RhoA/Rock1 pathway was less clear. Considering RBP4 was mainly a secretive protein, novel membrane receptors was possibly involved in promoting RBP4 signaling. Further studies were in demand to fully elucidating the RBP4 signaling pathways that related to cancers.

We observed that knockdown of RBP4 can greatly suppress ovarian cancer cell migration and proliferation. Considering RBP4 as a circulating protein, targeting RBP4 could be a relative easy option for ovarian cancer treatment, especially those associated with obesities.

## Conclusion

In conclusion, this study described the function of RBP4 in driving ovarian cancer cell migration and proliferation. Moreover, the underlying molecular mechanism of RBP4 was activation of RhoA/Rock1 pathway and CyclinD1 expression. Therefore, RBP4 could be a molecular bridge between obesity and cancers and a potential target for treating obese cancer patients.

## Abbreviations

RA: Vitamin A/retinol acid
RBP4: Retinol binding protein 4
STRA6: Stimulated by RA 6
TTR: Transthyretin
